# Assessing the Potential of LPWAN Communication Technologies for Near Real-Time Leak Detection in Water Distribution Systems

**DOI:** 10.3390/s21010293

**Published:** 2021-01-04

**Authors:** Michael Pointl, Daniela Fuchs-Hanusch

**Affiliations:** Institute of Urban Water Management and Landscape Water Engineering, Graz University of Technology, Stremayrgasse 10/I, 8010 Graz, Austria; fuchs-hanusch@tugraz.at

**Keywords:** water distribution system analysis, leak detection, low-power wide-area networks

## Abstract

While low-power wide-area network (LPWAN) technologies have been studied extensively for a broad spectrum of smart city applications, their potential for water distribution system monitoring in high temporal resolution has not been studied in detail. However, due to their low power demand, these technologies offer new possibilities for operating pressure-monitoring devices for near real-time leak detection in water distribution systems (WDS). By combining long-distance wireless communication with low power consumption, LPWAN technologies promise long periods of maintenance-free device operation without having to rely on an external power source. This is of particular importance for pressure-based leak detection where optimal sensor positions are often located in the periphery of WDS without a suitable power source. To assess the potential of these technologies for replacing widely-used wireless communication technologies for leak detection, GPRS is compared with the LPWAN standards Narrowband IoT, long-range wide area network (LoRaWAN) and Sigfox. Based on sampling and transmission rates commonly applied in leak detection, the ability of these three technologies to replace GPRS is analyzed based on a self-developed low-power pressure-monitoring device and a simplified, linear energy-consumption model. The results indicate that even though some of the analyzed LPWAN technologies may suffer from contractual and technical limitations, all of them offer viable alternatives, meeting the requirements of leak detection in WDS. In accordance with existing research on data transmission with these technologies, the findings of this work show that even while retaining a compact design, which entails a limited battery capacity, pressure-monitoring devices can exceed runtimes of 5 years, as required for installation at water meters in Austria. Thus, LPWAN technologies have the potential to advance the wide application of near real-time, pressure-based leak detection in WDS, while simultaneously reducing the cost of device operation significantly.

## 1. Introduction

The development of the Internet of Things (IoT), accelerating digitization and the consequently wide availability of low-power and low-cost communication technologies led to a continuous trend towards so-called smart cities and smart infrastructures. This trend was both induced and accompanied by the development of numerous communication protocols and modules for monitoring devices. Within the broad spectrum of wireless communication technologies and protocols theoretically suitable for smart city applications, from short-range technologies like Bluetooth, Wi-Fi or ZigBee [1] to long-range cellular technologies like 5G [2], only some standards combine low-power, long-range data transmission capabilities with transmissions rates suitable for most infrastructure monitoring applications. Alongside several highly energy-efficient radio technologies [3], such capabilities are offered by standards designed for ultra-low-power machine-to-machine (M2M) communication, among which low-power wide area network (LPWAN) standards, like Narrowband IoT (NB-IoT), Sigfox and long-range wide area network (LoRaWAN) are particularly promising for smart infrastructure monitoring [4]. 

Chaudhari et al. [5] and Mekki et al. [6] provide analyses of applications for LPWAN technologies, given the specific limitations of each communication standard. Listed applications range from traffic and infrastructure monitoring to smart rain barrels in [7]. However, while the authors consider a broad spectrum of use cases, to our knowledge no publication considers the advantages and drawbacks of these technologies in the context of data requirements for leak detection algorithms in water distribution systems (WDS). Such detection algorithms usually rely on data from hydraulic sensors, collected via pressure-monitoring devices and flow meters [8] operated at suitable positions in WDS [9,10], for model generation, calibration and validation. Once a detection algorithm is deployed, it relies on data streams from hydraulic sensors to identify leaks among deviations between modeled and monitored WDS behavior (e.g., [11]). Consequently, detection time and accuracy rely on these data streams.

Fast and reliable leak detection, based on real-time monitoring, can contribute to the reduction of water losses. In this context, Bonoli et al. [12] propose a framework for resource conservation based on green smart technology. Within this framework, Alvisi et al. [13] provide an extensive overview of IoT communication standards for open smart metering solutions in WDS, as well as hardware and software solutions for collecting and transmitting water meter readings with different communication protocols.

As many water utilities currently do not operate smart water meters or are in a state of transition during which conventional water meters are substituted by smart devices, they continue to rely on hydraulic sensors placed in their WDS for leak detection. However, as more and more inexpensive LPWAN monitoring devices become available and coverage of the corresponding networks is gradually expanded in both urban and rural areas, they allow water utilities to operate dense, large-scale sensor networks without the constraint of having to invest in their own communication infrastructure (e.g., gateways or signal concentrators).

Low-energy demand of LPWAN monitoring devices enables high sampling and data transmission rates without diminishing device runtimes. Consequently, by using NB-IoT, Sigfox or LoRaWAN, water utilities gain the possibility to generate time series of hydraulic and water quality parameters in high spatiotemporal resolution at relatively low operating costs, since, for instance, device maintenance efforts (e.g., regular battery changes) can be reduced considerably. 

Along with suitable time series databases and algorithms which are designed to analyze such time series in real-time, LPWAN technologies offer new possibilities in near real-time anomaly detection in WDS, e.g., to detect contamination events [14,15], cyber-attacks [16], as well as leaks and bursts [17,18,19].

Low energy consumption is of particular importance in the case of pressure-based leak detection where the optimal, most sensitive sensor positions are often located in the periphery of a WDS [10,20], where no external power source is available and energy-harvesting [21] might not be a viable option. Particularly in these cases, a key aspect of the operation of low-power monitoring devices is the trade-off between the device configuration (sampling and transmission rates) required for fast and reliable leak detection, the available battery capacity and required minimum device runtimes. 

In this work, the potential of LPWAN technologies to fulfil technical and operational long-term requirements of pressure monitoring for near real-time leak detection in WDS was analyzed. For this purpose, a conventional wireless wide-area network (WWAN) technology and the three above-mentioned LPWAN technologies were compared. Power consumption of a pressure-monitoring device, relying on GPRS (general packet radio service), a cellular (2G) standard for different sampling and data transmission rates, was assessed. In addition, an evaluation of how an alternative microcontroller (MCU), built around a communication module which is able to transmit data via NB-IoT, LoRaWAN and Sigfox, would influence power consumption was undertaken. Further, given the specifications and constraints of the three communication standards, the arising limitations for near real-time leak detection were derived. To ensure comparability, sufficient network coverage for either technology was assumed, as well as that the water utility uses existing communication networks, with all the possible drawbacks, like limitations due to fair access policies. 

For this assessment, a simplified, linear energy-consumption model was employed, combined with specifications from data sheets, laboratory-assessment and field-testing. This model was applied on common sampling and data transmission rates for leak and burst detection given in literature. 

This work is structured as follows. First, descriptions are provided for wide-area telecommunication technologies, data and device requirements for leak detection in WDS and an energy-efficient pressure-monitoring device, which was developed based on these specifications.

Second, the methodology of the conducted analyses is detailed, as well as the underlying assumptions for viable device configurations, power consumption and runtime analyses. Third, results of the comparison are presented and discussed. In the fourth and final section, conclusions and an outlook on further research, required to make full use of the potential of LPWAN technologies for near real-time leak detection in WDS, are offered.

## 2. Materials and Methods

### 2.1. Wireless Wide-Area Telecommunication Networks for WDS Monitoring

While energy-efficient data transmission is not a core problem for measurements at tanks or pumping stations of WDS, as they usually have an external power supply, the situation is entirely different for customer smart meters and pressure-monitoring devices within a WDS. In these cases, sensors with minimal power consumption have to be paired with energy-efficient communication technologies to allow maintenance-free operation without an external power source for multiple years. While most customer smart metering applications rely on highly energy-efficient radio technologies (e.g., versions of wireless M-Bus [13]) in combination with fixed network solutions [3], increasing coverage of LPWAN technologies [4,6,22] offers water utilities new possibilities to operate larger numbers of pressure-monitoring devices for fast leak detection.

Historically, many applications for long-range wireless communication for monitoring tasks were implemented by radio technologies or wireless broadband standards, like Wi-MAX or GPRS [1], for either security reasons or a lack of viable alternatives. As currently a transition towards nation-wide coverage for LPWAN standards can be observed, water utilities in urban and rural regions have the opportunity to decide between several technologies [23,24,25,26]. Each technology offers advantages and limitations, either based on operational constraints or technological and contractual requirements [13,27], which have to meet the standards for the data required by the applied detection algorithms. The expected performance and limitations of NB-IoT [28,29,30,31], Sigfox [32] and LoRaWAN [30,33,34,35,36,37,38,39] have been analyzed and tested extensively. In addition to energy consumption, the communication technologies vary regarding transmission rates (e.g., 100–600 bps for Sigfox versus 0.24–37.5 kbps for LoRaWAN), hourly or daily data transmission limits due to contractual terms or fair access policies in shared networks and expected transmission success or data loss rates. The last aspect is of particular importance when the sensors are installed in manholes or basements and even more so in densely-built urban environments, a use case, for which e.g., NB-IoT was specifically designed. 

Moreover, when deploying sensor networks in critical infrastructures like WDS, concerns of cyber security have to considered [40], as new threats and attack vectors are likely to arise with the wider use of LPWAN monitoring devices. Consequently, potential cyber risks resulting from IoT sensor networks need to be addressed with the same importance as the above-mentioned technological and operational advantages and limitations.

### 2.2. Data Requirements for Leak Detection in WDS

Aside from technical, contractual and organizational limitations of communication technologies, required sampling and data transmission rates, as well as minimum runtime requirements for monitoring devices in WDS have to be considered.

The spectrum of methods and algorithms applied to the problem of leak detection in WDS spans model-based, e.g., [41], data-driven [42] and combined approaches, e.g., [43]. Like the methodologies, the applied data sets to develop these approaches, train, and test the underlying algorithms vary. On the one hand, this is caused by the fact that different approaches use different monitoring principles for data generation, like flow [44], pressure [41], demand [17], acoustic signals [45], as well as combinations thereof [46]. On the other hand, in many cases where real data sets are used, multivariate time series representing normal operation and at times containing engineered leaks are used as provided by a water utility. While in other instances, simulated, artificial datasets, generated with a hydraulic model are applied [47], in some cases combined with data from engineered leaks for model calibration, validation and performance evaluation [43,46]. For data-driven approaches, data preparation and the structure of the provided data is of particular importance, as the quality of the underlying dataset is the basis for model development and performance [48]. While there are instances without a detailed description of the source of the applied dataset, its preparation or the initial exploratory data analysis, works like [49,50] provide a detailed description of their study site, sensor network and the preparation steps leading to the dataset used for algorithm development.

In [51] the authors assess the performance of different leak detection algorithms when providing data in sampling rates of 5 s, averaged to 1, 5 and 15 min. They conclude that no significant difference in detection performance can be observed between 1-min and 5-min sampling rates. In [52], Ye and Fenner consider the trade-offs in alarm rates and sampling rates by analyzing algorithm performance for sampling rates between 1 min and 30 min and conclude that higher sampling rates lead to faster burst detection. More recently, a similar study was published by Ahn and Jung [53], comparing performance changes of a hybrid statistical control method for burst detection when varying the number of sensors, as well as the sampling rate. Sampling rates of 5, 10, 15, 30 and 60 min are compared. These configurations can be found in multiple works on leak detection in WDS. For example, while the authors in [44] only consider flow data, the sampling rate in this case is 15 min with a data transmission rate of 30 min, similar to the authors of [17], who use a sliding window of 2 h, which is considered a substitute for the transmission interval. Other works use sampling rates between 1 min [54], 5 min [55,56,57,58], 10 min [59], or 15 min [11,60,61,62,63,64,65,66], which in one case were resampled [62]. Choi et al. [67] propose a Kalman-Filter-based methodology making use of adaptive sampling rates between 1 min and 1 h, which would directly impact sensor device runtime. This is of particular importance, as adaptive sampling rates might not only improve algorithm performance, but increase device runtime in the process. 

### 2.3. Operational Constraints for Low-Power Monitoring Devices in WDS

In addition to leak detection performance, minimum device runtime is an important constraint for water utilities when deploying battery-powered monitoring devices at WDS-scale. To facilitate the adaptation of near real-time leak detection with pressure-monitoring devices, operating costs have to match the generally low initial cost of LPWAN monitoring devices [6]. These devices have to run maintenance-free for long periods of time. Given an ideal, compact design, battery capacity is limited, which directly translates to the feasible device configurations, as high sampling and transmission rates limit the achievable runtimes. 

When installed at fire hydrants, a pressure-monitoring device has to be able to operate reliably for a minimum time span of several months between frost periods. Installation at house connections and water meters requires a minimum device runtime of several years with a single battery charge. An example for a minimum runtime requirement in this case is the interval for obligatory water meter calibration, which is currently five years in Austria [68].

For runtime analyses, configurations from literature on leak detection using pressure data or combinations of pressure, flow and/or demand data are applied. Since the overall goal is to evaluate the suitability of pressure-monitoring devices for near real-time leak detection, continuous operation is assumed. 

However, as online operation of devices and detection algorithms is in many cases only emulated in literature, with pre-processed data from static databases, sampling and especially transmission rates are not always discussed in detail. Thus, in order to provide an integrated analysis of energy consumption, a broad spectrum of sampling and transmission rates is considered in this work (see Section 2.7). To assess the achievable device runtime across LPWAN technologies, runtimes are compared for a compact battery with an available energy capacity of 3700 mWh, which is currently installed in our pressure-monitoring devices. Additionally, the above-mentioned minimum runtime of five years is used to derive battery sizes required to attain this runtime with device configurations for leak detection from literature.

### 2.4. Energy-Efficient Pressure-Monitoring Device

**Figure 1:**
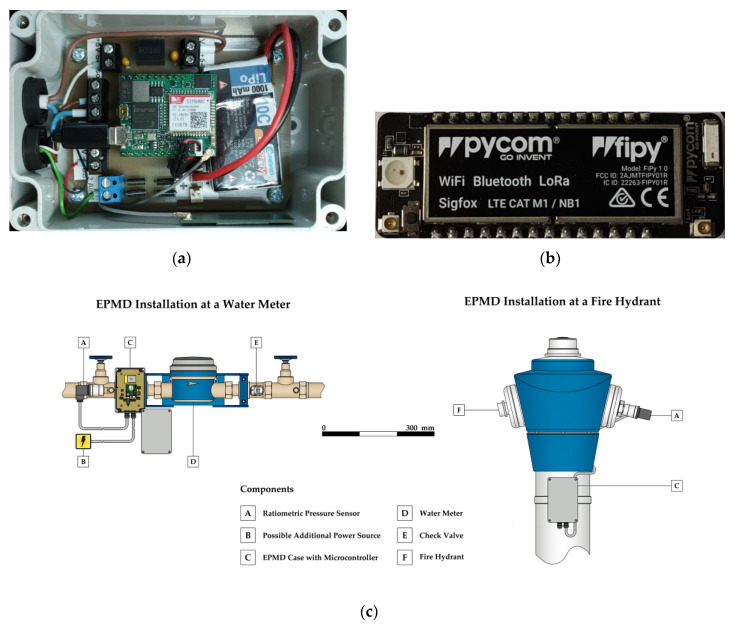
A microcontroller (MCU) with SIMCom SIM800C (general packet radio service, GPRS) communication module (**a**), MCU with Sequans Monarch (NB-IoT) and Semtech SX1272 (long-range wide area network, LoRaWAN and Sigfox) communication modules (**b**); schematic representations of energy-efficient pressure-monitoring device (EPMD) prototype installation at a water meter and at a fire hydrant (**c**).

For the research presented in this work, an all-purpose, energy-efficient pressure-monitoring device (EPMD), with an ultra-low-power microcontroller with a GPRS communication module (Figure 1a) was developed. The device is designed to meet the requirements of two use cases. First, it has to be able to operate temporarily at fire hydrants. Second, its design has to enable long-term installation at house connections in basements, inspection pits and manholes (Figure 1c). 

Given the relatively high energy consumption of GPRS compared to other communication technologies, the EPDM has an internal battery in a water and dust-proof case with an IP68-rating, but can be combined with any external power source, ranging from a larger battery to a common power outlet, solar panels or a micro turbine [69]. Additionally, the layout of the device’s circuitry allows the use of different microcontrollers (MCUs) for control and data transmission. While the device uses a rapidM2M M3 MCU [70] by company Microtronics in its GPRS-configuration, it relies on a FiPy MCU [71] by company Pycom (Figure 1b) for data transmission via NB-IoT, LoRaWAN and Sigfox. 

**Figure 2:**
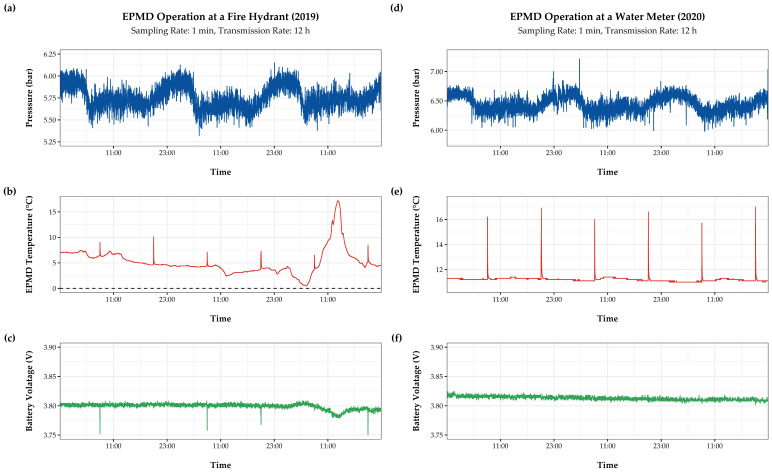
Snapshots from long-term field-testing of the EPMD at a fire hydrant (**a**–**c**) and a water meter (**d**–**f**).

The pressure sensors [72] of the EPMD, which use a linear voltage-signal-conversion to monitor pressure at a fixed input current, have an IP65-rating and all wetted surfaces are made from stainless steel, thus suitable for contact with drinking water. The devices are designed in a way that enables the use of an external antenna, if required. All cable glands are water and dustproof. Field tests showed that the devices can withstand temperatures below 0 °C, as well as above 30 °C, heavy rain, snow and even temporary submersion. Figure 2a–c shows a period of EPMD operation at a fire hydrant by the end of fall of 2019, where the device was exposed to all these weather changes and periods of direct insolation, with temperatures ranging between −5 °C and 25 °C. In comparison, in Figure 2d–f, an EPMD is operated at a water meter under more stable conditions in a basement of the same area in 2020. Pikes in temperature time series indicate data transmission.

### 2.5. Energy Consumption Model and Device Runtime Calculation

To assess energy consumption of the EPMD, a simplified energy consumption model is employed, which was derived from multiple sources on the calculation of GPRS [73], NB-IoT [22,74,75], LoRaWAN [22,76,77,78] and Sigfox [22,79] energy consumption. Simplifications are based on operational experience with the EPMD and recent publications including analysis and field testing of LPWAN sensors and monitoring devices [13,27].

The applied model is considered accurate enough for the assessment in this work, even though device performance and thus energy consumption can depend considerably on on-site conditions. The aim of using this simplified model is to provide a sufficiently accurate comparison, while ensuring comparability among the communication technologies.

**Figure 3:**
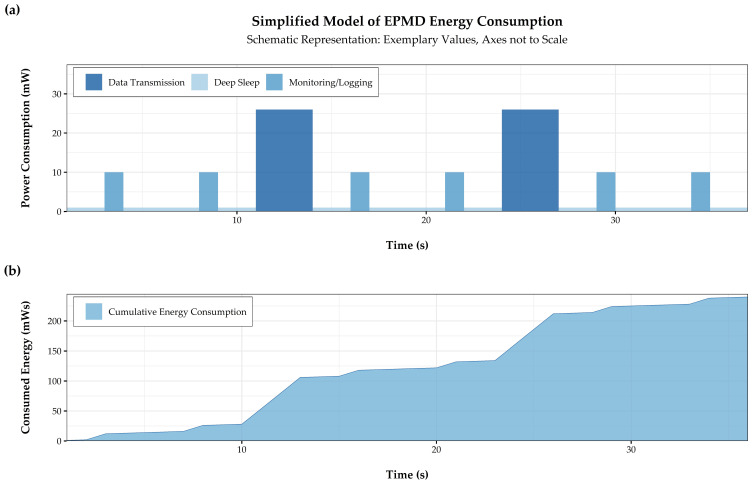
Simplified energy consumption model (**a**) with discrete events of mean power draw and translation to a cumulative energy consumption over time (**b**) for EPMD runtime analysis.

The model assumes that EPMD operation is a continuous sequence of the three basic operating states deep sleep, monitoring/logging and data transmission, as depicted in Figure 3a.

For this simplified model, energy consumption of the EPMD and its individual components (microcontroller, pressure sensor and GPRS communication module) were monitored for all operating states in multiple trial runs in the laboratory. Measured energy consumptions for the rapidM2M M3 MCU are simplified to mean consumptions for all operating states, as well as mean durations for the operating states monitoring/logging and data transmission (Figure 3a). In a similar manner, power consumption and transmission specifications for the three LPWAN technologies, where no measured data was available, were derived from the MCU datasheet [71] and findings of similar studies [22,79]. By employing this approach, it was possible to include the specifications for all four communication technologies. This allows for consideration of technology-specific effects for every operating state, which can vary considerably. 

Energy consumption during deep sleep depends primarily on the ability to shut down elements of the MCU, peripheral electronics and the pressure sensor, when not required, relying primarily on MCU configuration and energy efficiency. As indicated by its name, energy consumption of the sensor dominates the operating state monitoring/logging, with a neglectable consumption for mean calculation, binary encoding and storage of taken pressure samples. Laboratory tests with the ratiometric pressure sensor showed that two seconds of sensor operation, including device wake-up, high-frequency monitoring at several hundred Hertz and device shut-down, are sufficient to generate reliable readings. This sensor configuration is used for all following analyses.

Energy consumption for the operating state data transmission depends primarily on the selected communication technology and module. There are significant differences in the amounts of time and energy required for establishing a connection to the network, transmitting the data package, detaching from the network before going back to deep sleep. All analyses in this work, assume that EPMD operation is timed by an on-board real-time clock and communication modules of the MCUs are configured for unidirectional communication with uplink and data transmission, and confirmation downlink only if required. Additionally, technical and contractual limitations imposed by the respective communication technologies are observed.

As depicted in Figure 3b, daily energy is calculated according to Equation (1), for a number of potentially feasible configurations, meaning a combination of sampling and transmission rates, derived from the introductory literature review on leak detection in WDS. 

Daily energy consumption ($E_{sr,tr}^{tech}$) for a communication technology (*tech*), a sampling rate (*sr*) and a transmission rate (*tr*), is the sum of mean energy consumption and duration for all samples ($\underset{samples}{\sum }E_{sr}^{tech}\times t_{sr}^{tech}$) and transmission ($\underset{transmissions}{\sum }E_{tr}^{tech}\times t_{tr}^{tech}$) in a day, plus the energy consumption for the time the monitoring device is operated in deep sleep ($E_{ds,tech}\times t_{ds,tech}$). Based on daily energy consumptions derived with Equation (1), the EPMD’s runtime $(runtime_{tech,batt}$) for a fixed battery size ($E_{cap,batt}$) is calculated according to Equation (2).

(1) $$E_{sr,tr}^{tech}=\sum_{samples}E_{sr}^{tech}\times t_{sr}^{tech}+\sum_{transmissions}E_{tr}^{tech}\times t_{tr}^{tech}+E_{ds,tech}\times t_{ds,tech}$$

(2) $$runtime_{tech,batt}=\frac{E_{cap,batt}}{E_{sr,tr}^{tech}}$$

To assess possible runtime improvements when using LPWAN technologies, first the expected device runtime for the battery currently used in the device with an available energy capacity of 3700 mWh is calculated. Before second, the required energy capacity for a minimum device runtime requirement of five years is determined with the same equation.

Since efficient data encoding is a key factor for efficient operation of LPWAN technologies, uniformly encoded payloads are applied for all tested EPMD configurations in all energy consumption and runtime comparisons.

### 2.6. Payload Encoding

To compare energy consumption across communication technologies objectively, standardized payloads were defined, which are made up of uniform, binary encoded strings, representing a sequence of measurements by the EPMD for different ratios of sampling and transmission rates. The selected form of data encoding, as shown in Table 1, follows three basic assumptions. First, the EPMD is configured such that it calculates the average voltage of all measurements taken at multiple hundred Hertz over two seconds and provides a single pressure value in bars for each timestamp according to the selected sampling rate. The measurement generated has three decimal points, allowing to record pressure changes of 0.001 bars or one centimeter. Second, the resulting measurement is converted into a binary string (two’s complement) of two bytes. 

Missing values (e.g., in case of sensor malfunction) are encoded as a string of zeros. Resulting binary strings are stored into the internal memory of the EPMD. When the sampling rate exceeds the transmission rate, measurements are sequentially appended to the existing binary string without a delimiter character. 

Once successfully transmitted, binary payload strings are split in two-byte subsections and converted back to doubles representing the actual pressure measurements in bar. Individual measurements are reassigned a timestamp reflecting the time the sample was taken and stored in a suitable time series database. This uniform encoding allows the consideration of effects of technological and contractual constraints, as well as fair access policies and best practice guidelines for different sampling and transmission rates specific to the four communication technologies. This is of particular importance, given the strict package size limitations and number of daily transmissions for Sigfox, or device airtime limitations when using LoRaWAN.

### 2.7. Feasible EPMD Configurations

To assess the feasibility of the three LPWAN technologies for use in the EPMD and thereby for near real-time leak detection in WDS, 44 sampling and 44 transmission rates were selected, ranging from five seconds to four hours. The step width between sampling and transmission rates is increased as the temporal resolution increases (Table 2).

**Figure 4:**
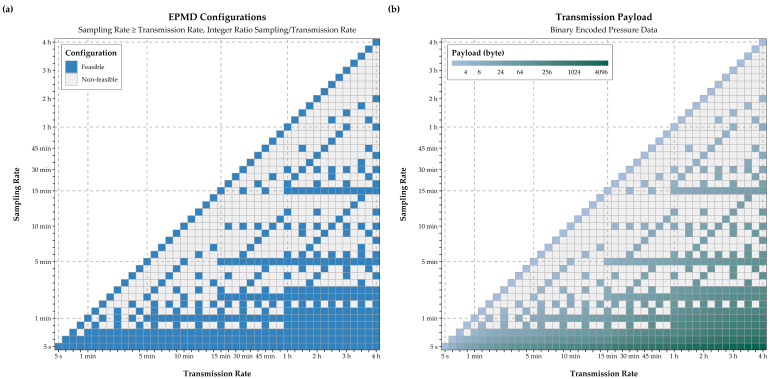
Feasible EPMD configurations (**a**) and payload size for a single transmission (**b**).

Among the potentially suitable configurations, those for which the transmission rate exceeds the sampling rate or those with a non-integral ratio of sampling and transmission rate were not considered. Derived feasible configurations are marked by blue tiles in Figure 4a, while the corresponding payload size per transmission, based on the above-mentioned encoding, is represented in the tiles in Figure 4b. 

For the EPMD configurations in Figure 4a, the feasibility was assessed individually for each communication technology, based on constraints of the energy consumption model, the encoding of the payload and transmission performance of the communication modules on the MCUs. Further, constraints and technology-specific limitations on payload or package size [22], as well as hourly device airtime limits or the maximum number of daily transmissions were considered. Since actual transmission rates may vary based on the conditions encountered at the installation site (e.g., inadequate network coverage), data transmission rates based on the values offered in the MCUs data sheets (Table 3) were assumed.

**Figure 5:**
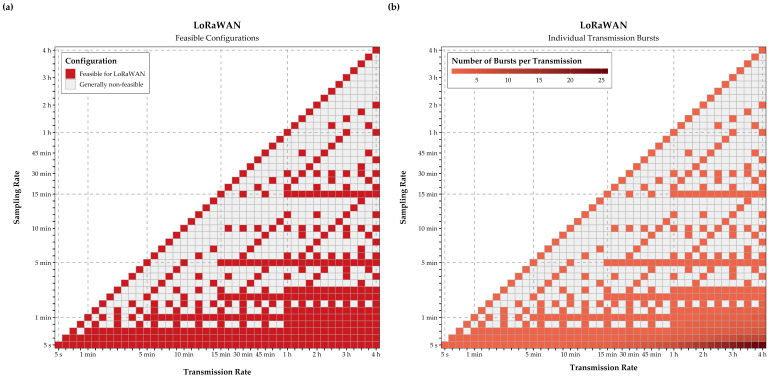
Transmission bursts (**b**) required to enable the implementation of configurations (**a**) with high sampling and low transmission intervals within the constraints of LoRaWAN.

These transmission rates were not only applied to calculate energy consumption for data transmission, but to verify whether a configuration violates policy or contractual limits of LoRaWAN or Sigfox. For LoRaWAN, specifications and limitations for a frequency of 868 MHz were assumed, as is standard in Austria [81], with a spreading factor SF 7 and a bandwidth of 250 kHz, leading to a maximum package size of 243 bytes and maximum application payload of 222 bytes. Consequently, configurations with high sampling and low transmission rates exceed this payload limitation for a single transmission. In these instances, a transmission was split into subsets of so-called bursts, a set of sub-transmissions with the maximum payload, minimizing overall device airtime. Figure 5b contains the number of bursts comprised by a single transmission.

**Figure 6:**
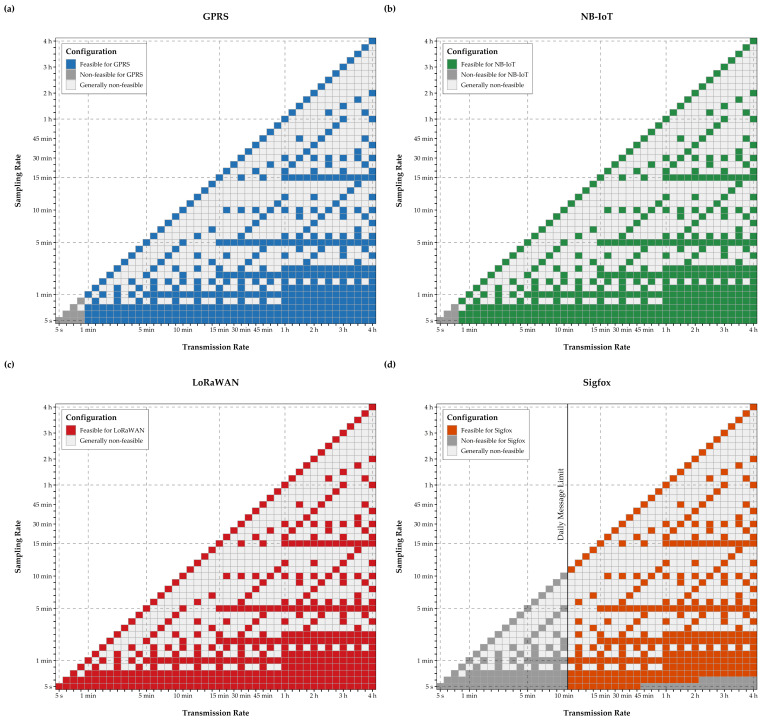
Feasible configurations for each communication technology (**a**–**d**).

For Sigfox, radio configuration RC1 and contractual limits were assumed, as offered by Austrian Sigfox operator Heliot [82], meaning a maximum payload size of 12 byte and a maximum number of 140 daily messages (see Figure 6d). For GPRS, transmission rates below one minute were not considered, as they would require permanent or quasi-continuous operation of the communication module in transmission state, thus violating the constraints of the energy consumption model and defying the purpose of low-power monitoring. For the same reason, transmission intervals under 45 s were disregarded for NB-IoT. Limitations on data transmission for NB-IoT at a frequency of 200 kHz can be contractual and specific to cellular network carriers. Nonetheless, the maximum payload size for a single NB-IoT transmission is limited to 1600 bytes [22], a constraint, which was considered by splitting respective transmissions into multiple subsequent bursts, similar to the approach for LoRaWAN. 

Given these assumptions and limitations, the final subset of feasible configurations was determined individually for GPRS, NB-IoT, LoRaWAN and Sigfox, represented by the tile colors in Figure 6. With the approach for splitting large transmissions into individual bursts, all device configurations can be analyzed for LoRaWAN. In contrast, the maximum of 140 daily transmissions and payload restrictions reduce the feasible configurations for Sigfox significantly. 

## 3. Results and Discussion

### 3.1. EPMD Operating States and Mean Energy Consumption

**Figure 7:**
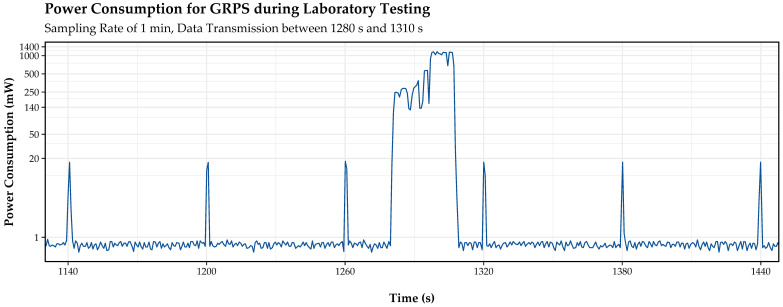
Energy-consumption time series for the EPMD with GPRS.

Figure 7 shows a power-consumption time series for GPRS as monitored with the EPMD in the laboratory. The time series depicts the power draw during deep sleep, interrupted with a peak every minute, indicating sensor operation and data logging on the internal storage of the device. The plateau occurring between seconds 1280 and 1310 is caused by data transmission. Power supply of the EPMD’s components is controlled by self-developed circuitry, regulating the voltage required by the sensor and the MCU when supplied from a battery. Energy losses arising from this circuitry were considered in the following analysis and results.

Results from device testing ensure that the pressure sensor provides stable results when operating the EPMD in state monitoring/logging for a maximum of 2 s. Given the low energy consumption of the MCUs, consumption for this operating state is solely based on the pressure sensor, which requires a mean input current of 4 mA and a maximum input current of 4.2 mA with a stable input voltage of 5 V (Table 4).

The stable base line between the peaks of Figure 7, which represents the operating state deep sleep, indicates that constant consumption for this operating state can be assumed. Based on laboratory testing and data from literature, the mean power consumptions for deep sleep of Table 4 were determined for the two MCUs.

Since, in contrast to the rapidM2M M3 MCU, laboratory testing of the FiPy MCU was not yet conducted, a set of definitions was introduced to ensure comparability across communication technologies, while facilitating the use of the simplified energy consumption model. These definitions are based on the two MCUs’ documentation, laboratory testing for GPRS and findings of recent studies investigating power consumption of LPWAN communication technologies. Accordingly, energy consumption for data transmission was split into (i) an overhead resulting from the processes of establishing and ending a connection to the communication network, which is specific to a communication technology and constant, regardless of data transmission rate, and (ii) the actual data transmission process, which depends on package size and transmission rate. Thus, the actual transmission process varies by technology and device configuration. 

EPMD tests show peak power consumptions of up to 1200 mW during data transmission via GPRS, as depicted between second 1280 and second 1310 in Figure 7. However, mean power consumption varies during this operating state due to fluctuations in the duration the device requires to establish a connection to the network. To account for this effect, a mean overhead of 26.5 s and a corresponding mean current during data transmission of 141 mA were derived for GPRS transmissions.

For NB-IoT, with the exception of a data transmission rate of 55 kbps, no energy consumption information is provided in the documentation of the FiPy MCU. Consequently, data and findings from [22], albeit for a different MCU and network service provider, were used as guide values for this technology. By using values and characteristics from a data transmission time series in this work, a rounded up overhead of 18 s and a mean current of 47 mA were assumed for the following analysis. In contrast to [22], configurable periods, during which the communication module would listen for downlink messages were not considered in our analysis.

As findings in [79] and [22] indicate, Sigfox power consumption for a single, unidirectional data transmission can vary, depending on the selected MCU and communication module. Given the dominant role of the actual data transmission for Sigfox energy consumption in both works, no overhead was assumed for transmissions with this technology.

Similar definitions were made for LoRaWAN. For transmissions with this technology, a Class A end-device with a mean supply current of 28 mA and a transmission power of 13 dBm [71] was assumed. Further, ideal conditions were presumed, during which the MCU only consumes deep sleep power of 0.1 mW while waiting for transmission acknowledgement by the gateway, which is received within the first transmission window [79]. Results in [78] and [22] show potentially significant variations in LoRaWAN energy consumption. These variations stem from the energy-efficiency of the MCU or the configuration of the communication module. Since they are specific to each use case, no overhead for LoRaWAN transmissions was selected. Nonetheless, depending on the employed communication module, the required reliability and range of transmissions, as well as the number of gateways or monitoring devices, necessary adaptations of LoRaWAN parameters might require incorporation of significant overheads, which can considerably increase energy consumption [76]. 

The resulting equations for calculating transmission times of all four communication technologies, measured power consumption values, as well as those derived from literature, were combined in Table 5.

While accounting for losses caused by the design of the device, all power draws and respective energy consumptions for the MCUs were calculated with a supply voltage of 3.7 V as provided by the battery, which, if required, can be further regulated by the circuitry of the EPMD. 

### 3.2. Daily Energy Consumption

Based on the introduced definitions for deep sleep, monitoring/logging and data transmission from the previous section, daily energy consumption for the four communication technologies was calculated according to Equation (1). 

**Figure 8:**
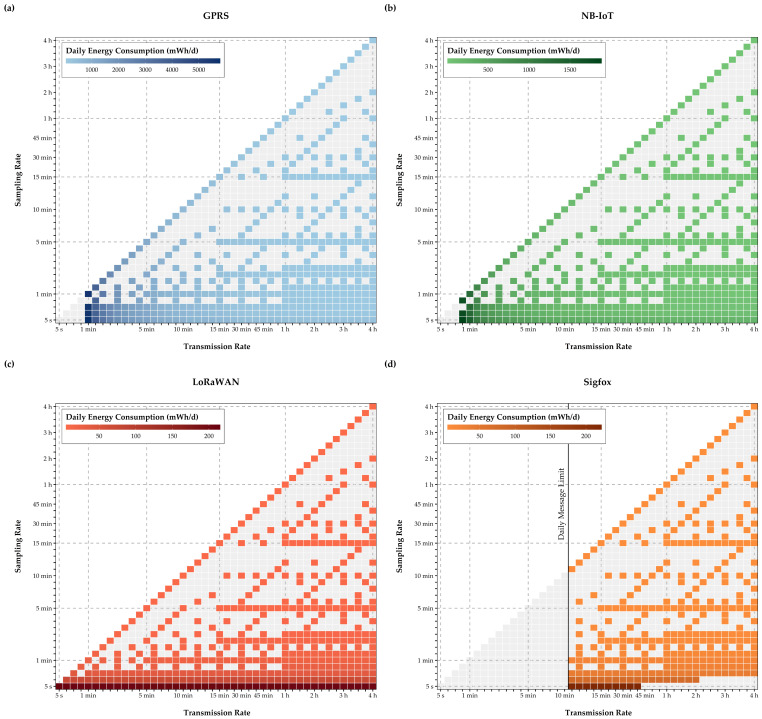
Daily energy consumption for feasible EPMD configurations (**a**–**d**).

As expected, results in Figure 8 point out the high energy demand of GPRS when compared to the three LPWAN technologies. GPRS energy consumption is almost three times as high as the daily demand of NB-IoT. Daily energy consumption of LoRaWAN and Sigfox is a decimal power lower than for NB-IoT. Nonetheless, the limitations of Sigfox become apparent in this figure, as no transmission rates below 10 min or larger package sizes are possible due to contractual limitations. However, even when considering these limitations, these results for daily energy consumption indicate that by using LPWAN communication technologies, improvements for EPMD device runtime between 20 and 30 times can be achieved.

### 3.3. EPMD Runtime Comparison

Based on daily energy consumptions from the previous section, Equation (2) was used to calculate and assess possible EPMD runtimes when using LPWAN technologies instead of GPRS. For this analysis, a battery with an available energy capacity of 3700 mWh was assumed, as such a model is currently installed in the pressure-monitoring device.

**Figure 9:**
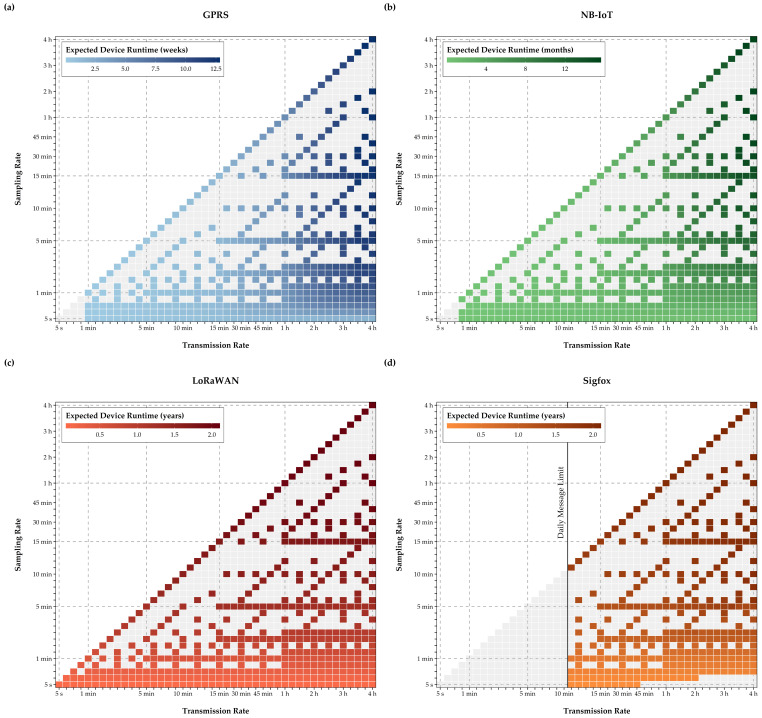
Expected EPMD runtime with a 3700 mWh battery (**a**–**d**).

Results in Figure 9 show that device runtime can be significantly improved when swapping GPRS for LPWAN communication. While the small battery limits EPMD runtimes with the GPRS-module to around three months under ideal on-site operating conditions, which, as filed tests showed, can be diminished to several weeks, without a battery change or an external power source, all other technologies offer runtimes exceeding one year. Nonetheless, runtimes of two years can only be achieved with sampling rates exceeding 15 minutes and data transmission rates over one hour for LoRaWAN and Sigfox. In comparison, with NB-IoT, even when reduction sampling and transmission rates, devices can barely exceed runtimes of one year with the currently used battery. Interestingly, the horizontal color gradients in Figure 9 show the influence of transmission energy consumption for GPRS and NB-IoT, where longer runtimes require a transmission rate beyond one hour. In contrast, the vertical color gradient for LoRaWAN and Sigfox indicates that power consumption for monitoring and data logging on the MCU drives overall energy consumption.

**Figure 10:**
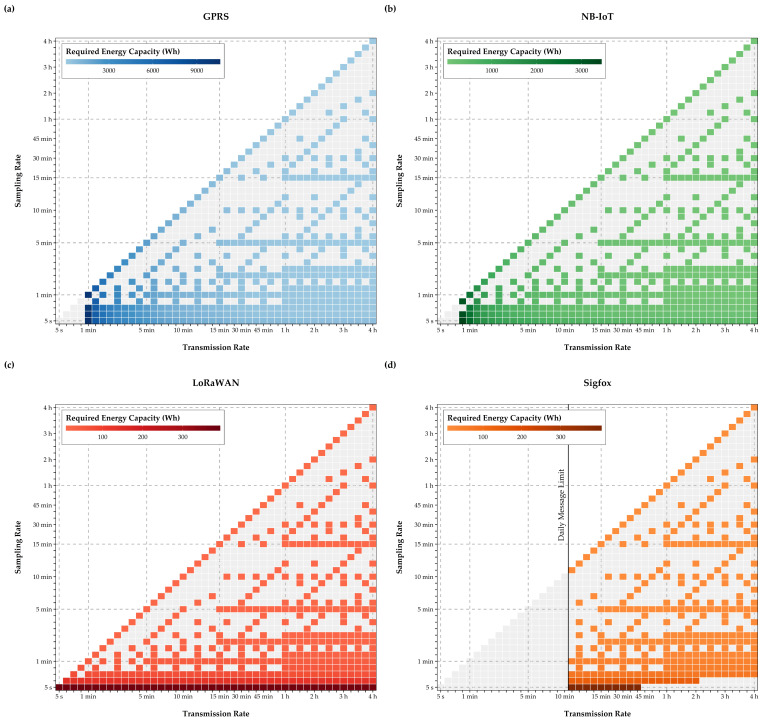
Expected battery capacity for five years of maintenance-free EPMD runtime (**a**–**d**).

In order to determine which energy capacity is required to operate the EPMD at water meters for a minimum period of five years, which is the current mandatory interval for water meter calibration or replacement in Austria, this runtime requirement and daily energy consumptions were combined via Equation (2). The results of this calculation are represented in Figure 10. 

This analysis clearly indicates that long-term operation of a GPRS module in the EPMD without an external power source is not feasible for long-term operation (e.g., for five years at a water meter). Even low sampling and transmission rates would require a sizeable battery or an external power source, like a solar panel, or some form energy harvesting, like a micro turbine. The required energy for NB-IoT is considerably smaller, and less than a third of GPRS’ requirements for sampling and transmission rates around one minute, which would be an interesting configuration for near real-time leak detection in WDS. While such configurations are not possible for Sigfox, LoRaWAN has the potential to implement leak detection with high-frequency measurements and a compact battery. For both, Sigfox and LoRaWAN, the required energy capacity for a device runtime of five years is an order of magnitude smaller than for NB-IoT.

LoRaWAN can meet the five-year runtime requirement with the highest sampling and transmissions rates of this analysis, while retaining a batter capacity, which is smaller than the one required by GPRS for a four-hour sampling and transmission rate.

Consequently, a common sampling rate of five minutes and a transmission rate of 15 minutes for leak detection can be realized with relatively small batteries for LoRaWAN and Sigfox, thereby ensuring a compact EPMD design, while enabling flexible deployment (e.g., installation in confined spaces like inspection pits), without increasing maintenance requirements for battery changes.

### 3.4. Implications for WDS Monitoring and Near Real-Time Leak Detection

As the results of this work indicate, EPMD-runtime gains derived from applying LPWAN communication technologies instead of GPRS can be considerable. In accordance with the findings of [1] or [22], the potential of LPWAN technologies for reliable, energy-efficient data transmission, extending device runtimes, without having to rely on external power sources or frequent battery changes, is considerable. Even though calculations with the introduced energy-consumption model are based on certain simplifications and assume optimal operating conditions, it is reasonable to infer that the EPMD will be able to sustain sampling and transmission rates between five and 30 min, commonly used in literature on leak detection in WDS [51,52,53] with LPWAN data transmission. LoRaWAN in particular has the potential to meet the defined five-year runtime requirement without sacrificing the device’s compact design with a small internal battery. 

Nonetheless, LPWAN transmission performance can be adversely affected during deployment in WDS under real-world conditions, diminishing potential runtime increases and the spectrum of feasible sampling and transmission rates. 

In addition to regional variations of NB-IoT, Sigfox or LoRaWAN coverage, inadequate quality of service, local interference from building stock or vegetation, as well as effects of ambient conditions (e.g., variations in temperature) might lead to an increase in energy consumption or prohibit reliable operation. To that effect, numerous publications [29,38,39] discuss the specific limits of the compared technologies. Further, results in [13] or [27] confirm the influence of on-site conditions on transmission performance. Such effects, like higher energy consumption during periods of low temperature (e.g., see Figure 2c) and data losses at locations where transmission was impaired by cast iron covers, were repeatedly observed during EPMD field-testing. 

Moreover, national contractual and technological constraints for device configurations, for NB-IoT and Sigfox, as well as for LoRaWAN, which have not been considered in detail in the presented analysis, can severely reduce achievable transmission rates and device runtimes in real-time WDS monitoring. MCU selection can influence energy consumption across all operating states, while potentially necessary adjustments to device configurations, for example, to accomplish long-distance transmissions, can reduce transmission performance. 

Thus, all the above-mentioned aspects have to be considered, when selecting the most suitable LPWAN technology, since this selection does not only determine the achievable data quality for leak detection and required battery capacity. It also influences the initial cost of monitoring devices and communication infrastructures [4,6], as well as labor costs for device maintenance (e.g., battery changes) and operating costs for data transmission for cellular technologies or Sigfox. 

Nonetheless, even when considering potential adverse effects, the results of this work show the enormous potential of NB-IoT, Sigfox and LoRaWAN for maintenance-free, long-term pressure monitoring of WDS in near real-time. Moreover, by facilitating fast MCU changes, the EPMD’s modular design allows instant transitions between GPRS and LPWAN communication, allowing configurations tailored to specific monitoring tasks. 

While the presented analysis focused on instances where the utility does not operate its own communication infrastructure, flexible middleware solutions, like the one developed by Alvisi et al. [27], introducing a standardized framework for simple integration of different cellular and LPWAN technologies, can help to optimize pressure-monitoring networks for near real-time leak detection in WDS.

In addition to performance and cost, a key aspect to consider when implementing LPWAN sensor networks in critical infrastructures like WDS are cyber risks. While IoT sensor networks driven by LPWAN communication technologies introduce new possibilities for intrusion detection in industrial control systems (ICS) of water utilities, new treats and attack vectors arise with their implementation. Respective risks are likely to increase as LPWAN technologies see wider application in WDS monitoring and control. To mitigate risks while making use of the potential of these technologies, security gaps have to be addressed [40], guidelines have to be developed and awareness among ICS operators in the water sector has to be raised.

## 4. Conclusions and Outlook

LPWAN communication technologies offer new opportunities for long-term, maintenance-free operation of pressure-monitoring devices for leak detection in water distribution systems (WDS). In this work, the potential of such technologies as alternatives for data transmission in a self-developed, energy-efficient pressure-monitoring device (EPMD) was assessed. A device, which is designed to operate microcontrollers with either a GPRS or a combined NB-IoT, Sigfox and LoRaWAN communication module. An integrated assessment was provided, considering technological and contractual limitations of the four communication standards, as well as operational requirements for large-scale sensor networks in WDS, like easy and flexible installation, a compact design and long-term operation without an external power source. Paired with a literature review on leak detection and the algorithms and datasets used in these works, a simplified energy consumption model was used to calculate energy consumption and device runtimes with a compact battery. Further, the required battery capacities for a minimum device runtime of five years, as required for EPMD operation at water meters in Austria, was determined.

While the results of this work confirm findings from other research, showing the unique capabilities of LPWAN technologies for energy-efficient long-distance data transmissions, extensive field testing is required to quantify the impact of site-specific constraints, e.g., the availability and quality of service of LPWAN networks, as well as the impact of building stock on EPMD performance. Since these constraints are expected to have an impact on device runtime, as well as sampling and transmission rates, which can be achieved and reliably sustained, such analyses are not only considered to have merit, but to be necessary to determine the actual improvements LPWAN data transmission can provide for near real-time leak detection in WDS. 

Nonetheless, LPWAN communication modules allow water utilities to design and configure pressure-monitoring devices, tailored to their specific monitoring requirements. Even when keeping drawbacks due to sub-optimal operating conditions in mind, the presented findings indicate that LPWAN technologies enable device runtimes of multiple years for the compact EPMD, significantly reducing efforts for device maintenance without diminishing detection performance. While this is true for sampling and transmission rates currently used in leak detection, further research has to determine improvements for near real-time monitoring of WDS, as LPWAN data transmission enables the efficient collection and transmission of hydraulic data in even higher spatiotemporal resolution. However, given the potential of e.g., artificial neural network architectures for accurate and fast anomaly detection with high volumes of noisy and partially faulty sensor data, devices with LPWAN capabilities can be expected to advance real-time WDS monitoring on a larger scale, well beyond leak detection.

## Figures and Tables

**Table 1:** Example for binary encoding of monitored pressure data.

EPMD Configuration			Raw Data in bar	Representation for Encoding	Binary Encoded Measurements	Payload Size
Transmission Rate	Sampling Rate	Ratio				
30 s	30 s	1	6.125	06125	0001011111101101	2 byte
15 min	5 min	3	5.295 5.169 5.225	05295 05169 05225	0001010010101111 0001010000110001 0001010001101001	6 byte

**Table 2:** Configurations for sampling and data transmission rates with varying step widths.

Sampling or Transmission Rate	Step Width
5 s, 15 s, 30 s, 45 s, 1 min	10 s, 15 s
1 min–5 min	30 s
5 min–30 min	1 min
30 min–1 h	5 min
1 h–4 h	15 min

**Table 3:** Data transmission rates for the communication modules of the compared MCUs [71,80].

MCU	Communication Module	Communication Standard	Data Transmission Rate
rapidM2M M3	SIMCom SIM800C [80]	GPRS	85.6 kbps
FiPy [71]	Sequans Monarch	NB-IoT	55 kbps
	Semtech SX1272	Sigfox	100 bps
		LoRaWAN	1.1 kbps

**Table 4:** Energy consumption during deep sleep and monitoring/logging.

Operating State	MCU	Mean PowerConsumption	Based On
deep sleep	rapidM2M M3	0.75 mW	EPMD testing
	FiPy	0.1 mW	EPMD circuitry testing and [71]
monitoring/logging	rapidM2M M3	20 mW	EPMD testing
	FiPy	20 mW	EPMD circuitry testing with pressure sensor and [71]

**Table 5:** Assumptions for power consumption during data transmission.

Communication Standard	Transmission Time	Mean PowerConsumption	Based On
GPRS	26.5 s overhead + package size/86.5 kbps	521.7 mW	EPMD testing, [80]
NB-IoT	18 s overhead + package size/55 kbps	173.9 mW	EPMD circuitry testing, [22,71]
Sigfox	package size/100 bps	155.4 mW	EPMD circuitry testing, [22,71,79]
LoRaWAN	package size/1.1 kbps	103.6 mW	EPMD circuitry testing, [22,71,76,78]

## Data Availability

The data presented in this study are available on request from the corresponding author.
